# Maternal Dietary Docosahexaenoic Acid Alters Lipid Peroxidation Products and (n-3)/(n-6) Fatty Acid Balance in Offspring Mice

**DOI:** 10.3390/metabo9030040

**Published:** 2019-03-01

**Authors:** Bo Yang, Runting Li, Taeseon Woo, Jimmy D. Browning, Hailong Song, Zezong Gu, Jiankun Cui, James C. Lee, Kevin L. Fritsche, David Q. Beversdorf, Grace Y. Sun, C. Michael Greenlief

**Affiliations:** 1 Department of Chemistry, University of Missouri, Columbia, MO 65211, USA; bykm5@mail.missouri.edu; 2 Biochemistry Department, University of Missouri, Columbia, MO 65211, USA; lirun@health.missouri.edu (R.L.); sung@missouri.edu (G.Y.S.); 3 Departments of Radiology, Neurology and Psychological Sciences, and the Thompson Center, University of Missouri, Columbia, MO 65211, USA; twfnf@mail.missouri.edu (T.W.); beversdorfd@health.missouri.edu (D.Q.B.); 4 Department of Nutrition and Exercise Physiology, University of Missouri, Columbia, MO 65211, USA; browningj@missouri.edu (J.D.B.J.); fritschek@missouri.edu (K.L.F.); 5 Department of Pathology and Anatomical Sciences, University of Missouri, Columbia, MO 65211, USA; hskk8@mail.missouri.edu (H.S.); guze@health.missouri.edu (Z.G.); cuij@health.missouri.edu (J.C.); 6 Department of Bioengineering, University of Illinois at Chicago, Chicago, IL 60607, USA; leejam@uic.edu

**Keywords:** diet and dietary lipids, arachidonic acid, omega-3 fatty acids, lipid peroxidation, 4-hydroxyhexenal, 4-hydroxynonenal, autism spectrum disorder, liquid chromatography, tandem mass spectrometry

## Abstract

The abundance of docosahexaenoic acid (DHA) in the mammalian brain has generated substantial interest in the search for its roles in regulating brain functions. Our recent study with a gene/stress mouse model provided evidence to support the ability for the maternal supplement of DHA to alleviate autism-associated behavior in the offspring. DHA and arachidonic acid (ARA) are substrates of enzymatic and non-enzymatic reactions, and lipid peroxidation results in the production of 4-hydroxyhexenal (4-HHE) and 4-hydroxynonenal (4-HNE), respectively. In this study, we examine whether a maternal DHA-supplemented diet alters fatty acids (FAs), as well as lipid peroxidation products in the pup brain, heart and plasma by a targeted metabolite approach. Pups in the maternal DHA-supplemented diet group showed an increase in DHA and a concomitant decrease in ARA in all brain regions examined. However, significant increases in 4-HHE, and not 4-HNE, were found mainly in the cerebral cortex and hippocampus. Analysis of heart and plasma showed large increases in DHA and 4-HHE, but a significant decrease in 4-HNE levels only in plasma. Taken together, the DHA-supplemented maternal diet alters the (n-3)/(n-6) FA ratio, and increases 4-HHE levels in pup brain, heart and plasma. These effects may contribute to the beneficial effects of DHA on neurodevelopment, as well as functional changes in other body organs.

## 1. Introduction

The high abundance of docosahexaenoic acid (22:6n-3, DHA) in the phospholipids in brain and retina has led to an interest in the search for its functional roles in health and diseases [1]. DHA, a fatty acid with six carbon-carbon double bonds, is particularly important during brain development, as there is a “DHA accretion spurt” during the late gestational period, a time of rapid neurogenesis [2]. There is strong support for the role of n-3 polyunsaturated fatty acids (PUFAs) as essential nutrients for the brain [3], and mothers with low intake of n-3 PUFAs may show impairment in neurogenesis and contribute to neuropsychiatric disorders in the offspring, including increased risk of autism [4,5]. Maternal consumption of seafood and DHA supplementation during pregnancy was shown to improve behavior in offspring [6,7,8]. Despite positive results with animal models, studies with DHA supplementation on children with autism spectrum disorder have not provided consistent results. One possible explanation is due in part to delayed diagnosis of the disorder, and recommendation of n-3 PUFA treatment at a time too late in development to have an obvious positive impact [9,10,11]. 

Since understanding the mechanism(s) whereby dietary DHA offers beneficial effects to the human brain is still elusive, recent studies have resorted to answering this question using animal models. There is evidence that maternal diets enriched in n-3 PUFAs can promote neuronal development and synaptic function, alter mental activities, and even modify contents of neurotransmitters, including gamma-aminobutyric acid, dopamine, glutamate, and serotonin, and their metabolites [12,13,14]. Other studies support the ability for DHA to mediate the resolution of inflammation through the production of lipid mediators, such as resolvins and neuroprotectins [15,16]. In a gene/stress mouse model in which autistic behavior was associated with maternal deletion of one copy of the serotonin transporter gene [17], maternal dietary DHA was shown to alter dopamine and mitigate autism-associated behaviors in the pups [13,18]. In fact, the relative deficiency of DHA during pregnancy was sufficient to cause autism-associated behaviors in the pups regardless of whether or not maternal stress was present during pregnancy [18]. In an additional study, dietary DHA administered to mice exposed to a viral mimetic, polyriboinosinic-polyribocytidilic acid, during the gestational period, was shown to alleviate autism-associated behavior in pups [19]. Taken together, these studies are in agreement with the potential beneficial effects of dietary DHA to ameliorate neurobehavioral deficits, due to maternal stress or insults. 

**Figure 1:**
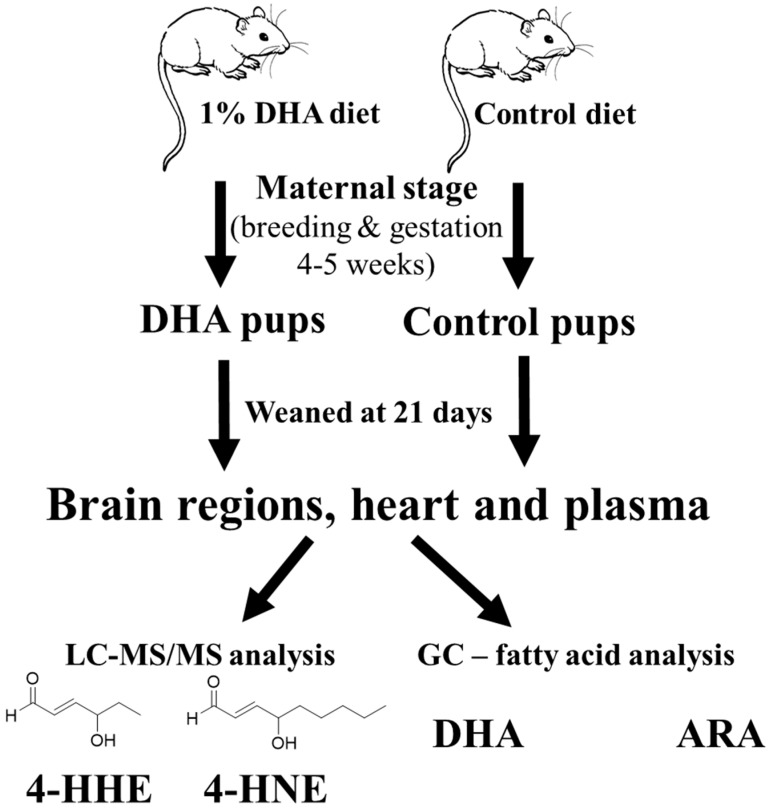
A scheme depicting the experimental protocol for 1% docosahexaenoic acid (DHA) and control diets to pregnant mice.

DHA, as well as arachidonic acid (20:4 n-6, ARA), are two major PUFAs that undergo enzymatic reactions to produce active lipid mediators [1]. These fatty acids (FAs) can also undergo non-enzymatic reactions through interaction with oxygen radical species from the environment, as well as other oxidative mechanisms. Lipid peroxidation may target PUFAs in the cell membranes, as well as in the free form, releasing 4-hydroxyhexenal (4-HHE) from DHA and 4-hydroxynonanal (4-HNE) from ARA. These alkenal products are readily detected in biological tissues and plasma [20]. In fact, the increase in production of 4-HNE has been regarded as a marker for oxidative stress in brain injury and stroke [21,22], as well as in infectious brain diseases, such as spirochete-induced encephalitis [23,24]. Depending on conditions and the amount released, 4-HNE can alter cell signaling pathways and undergo cell metabolism by forming adducts with proteins, DNA and lipids [25,26,27]. Although less is known about 4-HHE, there is evolving evidence that this alkenal compound also possesses properties similar to 4-HNE [28]. In a study by Nakagawa et al., dietary fish oil given to adult mice was shown to alter levels of the peroxidation products which were correlated with changes in FA composition [29]. However, information on lipid peroxidation products and FA composition in the brain of offspring mice nursed by mothers fed a DHA diet has not been examined in detail. In this study, pregnant mice were administered a control or a 1% DHA diet throughout gestation and lactation, and the levels of 4-HHE and 4-HNE in different regions of weaning pup brain were determined by liquid chromatography-tandem mass spectrometry (LC-MS/MS), and the FA composition was determined by gas chromatography (GC). In addition, this study also examined the effects of maternal DHA diet on 4-HHE and 4-HNE levels and FA composition in the heart and plasma. Figure 1 outlines the general protocol used in this study.

## 2. Results

**Figure 2:**
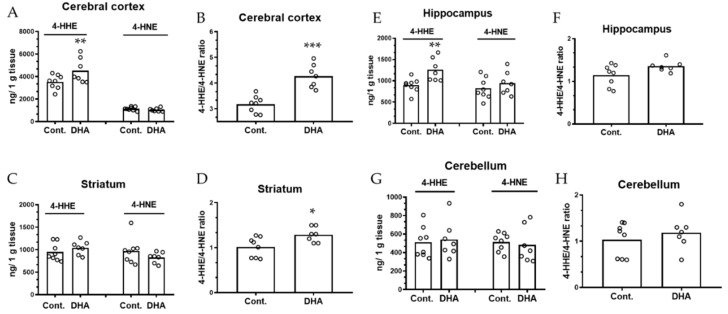
Maternal DHA intervention alters the levels of -hydroxyhexenal (4-HHE) and 4-hydroxynonenal (4-HNE) and 4-HHE/4-HNE ratios in offspring brain regions. (**A**,**C**,**E**,**G**): Levels of 4-HHE and 4-HNE in different brain regions (cortex, striatum, hippocampus, and cerebellum, respectively) from pups nursed by control and 1% DHA mothers. (**B**,**D**,**F**,**H**): Ratios of 4-HHE/4-HNE in different brain regions (cortex, striatum, hippocampus, and cerebellum, respectively). Levels of 4-HHE and 4-HNE in brain regions were measured using LC-MS/MS as described in text, and normalized to tissue weight. Each value represents the mean ± SEM of eight and seven pups from control and 1% DHA diet groups, respectively. Analysis using two-tail unpaired t-test indicated significance between DHA group and controls. * *p* < 0.05; ** *p* < 0.01; *** *p* < 0.001.

The levels of 4-HHE and 4-HNE were measured in different regions of the brain in the developing pups nursed by mothers given the control and DHA diets (*n* = 8 and 7 pups), respectively. For analysis of brain regions, the left cerebral cortex and left striatum were used but the left and right hippocampi were combined, and the cerebellum was not separated. For the initial analysis of 4-HHE and 4-HNE in the cortex, a tissue weight:water ratio of 1:5 (*w*/*v*) was used for processing the tissue. As shown in Figure 2A, levels of 4-HHE and 4-HNE in the cerebral cortex in control pups were 3528 ± 191 and 1146 ± 53 ng/g tissue, respectively (mean ± SEM), and 4531 ± 305 and 1050 ± 45 ng/g tissue in the DHA group, respectively. As for the striatum, hippocampus, and cerebellum, we used a tissue weight:water ratio of 1:8 (*w*/*v*) in order to allow more aqueous extract for analysis. Maternal dietary DHA also increased levels of 4-HHE in the hippocampus, but no significant changes were observed in the striatum and cerebellum (Figure 2C,E,G). These results also show no significant changes in levels of 4-HNE in any of the brain regions (Figure 2A,C,E,G). A significant increase in 4-HHE/4-HNE ratio was observed in the cortex and striatum of DHA pups as compared with control pups (Figure 2B,D, *p* < 0.05). A small but non-significant increase in 4-HHE/4-HNE ratio was also observed in the hippocampus. 

**Figure 3:**
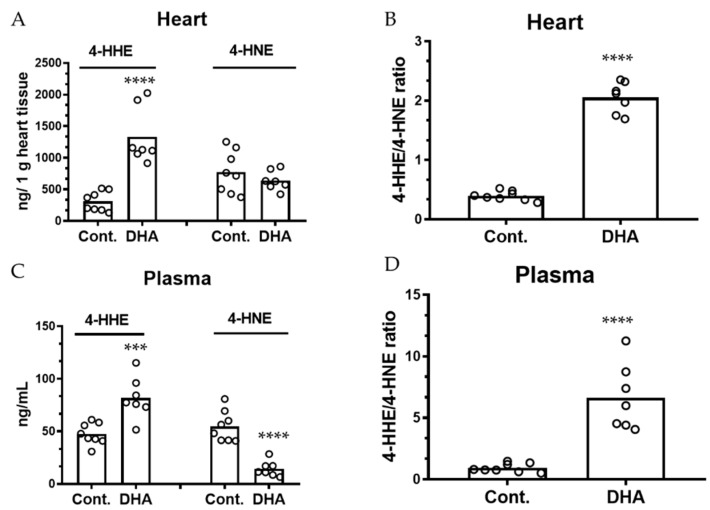
Maternal DHA intervention alters the levels of 4-HHE and 4-HNE and 4-HHE/4-HNE ratios in offspring heart and plasma. (**A**,**C**): Levels of 4-HHE and 4-HNE in heart and plasma of pups nursed by control and 1% DHA mothers, respectively. (**B**,**D**): Ratios of 4-HHE/4-HNE in hearts and plasma, respectively. Levels of 4-HHE and 4-HNE were measured using LC-MS/MS and normalized to tissue weight. Each value represents the mean ± SEM of eight and seven pups from control and 1% DHA diet groups, respectively. Analysis using two-tail unpaired t-test indicated significance between DHA group and controls. *** *p* < 0.001; **** *p* < 0.0001.

The levels of 4-HHE and 4-HNE in the heart tissue were 312 ± 55 and 774 ± 118 ng/g tissue, respectively, in the control group, and 1331 ± 168 and 642 ± 58 ng/g tissue, respectively, in the DHA pups (Figure 3A). These results led to a 5.2-fold increase of 4-HHE/4-HNE ratio in the heart when comparing the DHA pups with controls (Figure 3B, *p* < 0.0001).

In this study, a 30 µL plasma sample was obtained from each pup for measurement of 4-HHE and 4-HNE levels. In the control group, the concentrations of 4-HHE and 4-HNE in plasma were 47.6 ± 3.6 and 54.9 ± 5.2 ng/mL, respectively (Figure 3C). In the DHA diet group, concentrations of 4-HHE and 4-HNE in plasma were 81.9 ± 7.5 and 14.3 ± 2.8 ng/mL, respectively (Figure 3C). Plasma samples were the only ones showing a significant decrease in levels of 4-HNE in the DHA group. The significant (*p* < 0.0001) increase in 4-HHE and decrease in 4-HNE in the plasma resulted in a 7.1-fold difference in the 4-HHE/4-HNE ratios when comparing the pups nursed by DHA mothers with controls (Figure 3D, *p* < 0.0001).

**Figure 4:**
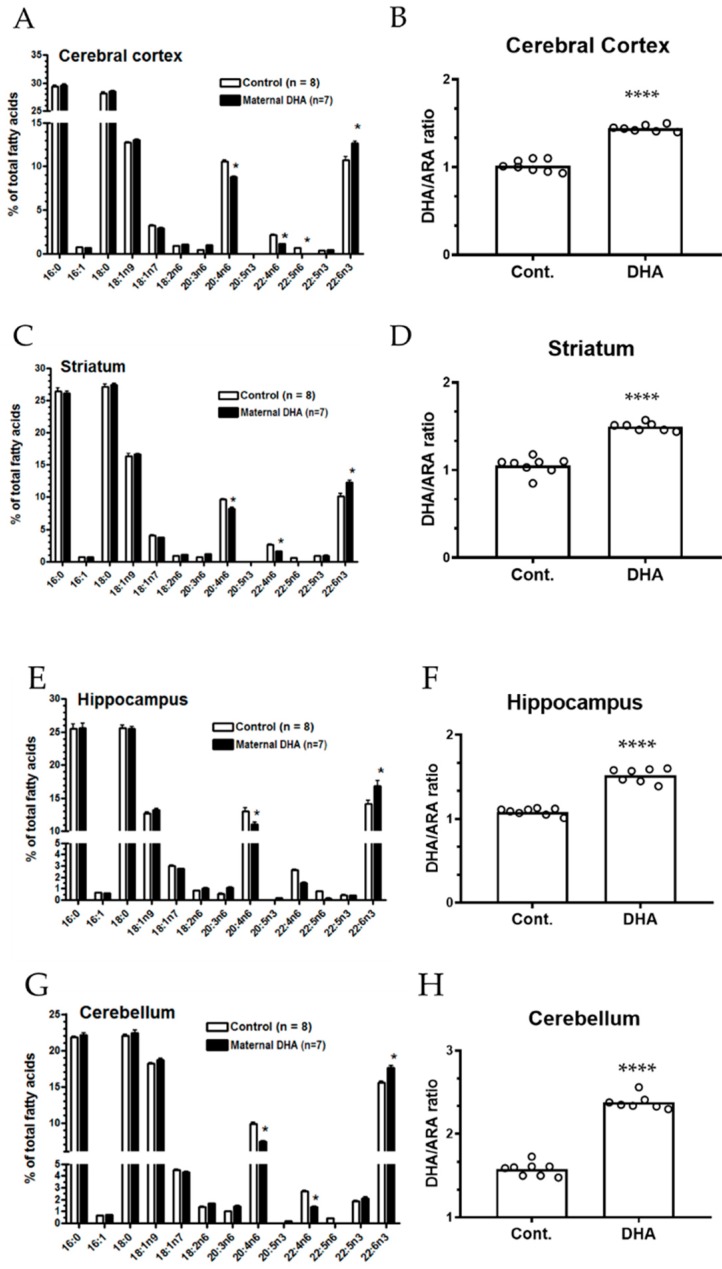
Maternal DHA intervention alters the fatty acid (FA) composition in offspring brain regions. (**A**,**C**,**E**,**G**): FA composition from different brain regions (cortex, striatum, hippocampus, and cerebellum, respectively) of developing pups nursed by mothers fed a control and 1% DHA diet. (**B**,**D**,**F**,**H**): Ratios of DHA/ arachidonic acid (ARA) in different brain regions (cortex, striatum, hippocampus, and cerebellum, respectively). FA composition was reported as percent of total FA content (g per 100 g). Each value represents the mean ± SEM of eight and seven pups from control and 1% DHA diet groups, respectively. Analysis of FA using one way-ANOVA indicated significance between 1% DHA diet group and controls. * *p* < 0.05. Analysis of DHA/ARA ratios using two-tail unpaired t-test indicated significance between DHA group and controls. **** *p* < 0.0001.

A summary of the FA composition of all brain regions, heart and plasma is presented in Supplemental Tables S1 and S2. Similar to our early studies [30], FAs in the brain tissue are characterized by low levels (less than 1%) of 18:2 (n-6) and higher levels (~10%) of 20:4 (n-6) and 22:6 (n-3). Analysis of FAs in all brain regions, including the cerebral cortex, hippocampus, striatum and cerebellum indicated small but significant increases in 22:6 (n-3) and decreases in 20:4 (n-6) and other n-6 FAs in the DHA pups as compared to the control group (Figure 4A,C,E,G, *p* < 0.05). A comparison of the 22:6 (n-3)/20:4 (n-6) (DHA/ARA) ratio indicated significant increases in all the brain regions when comparing maternal DHA pups with the control pups (Figure 4B,D,F,H, *p* < 0.0001).

**Figure 5:**
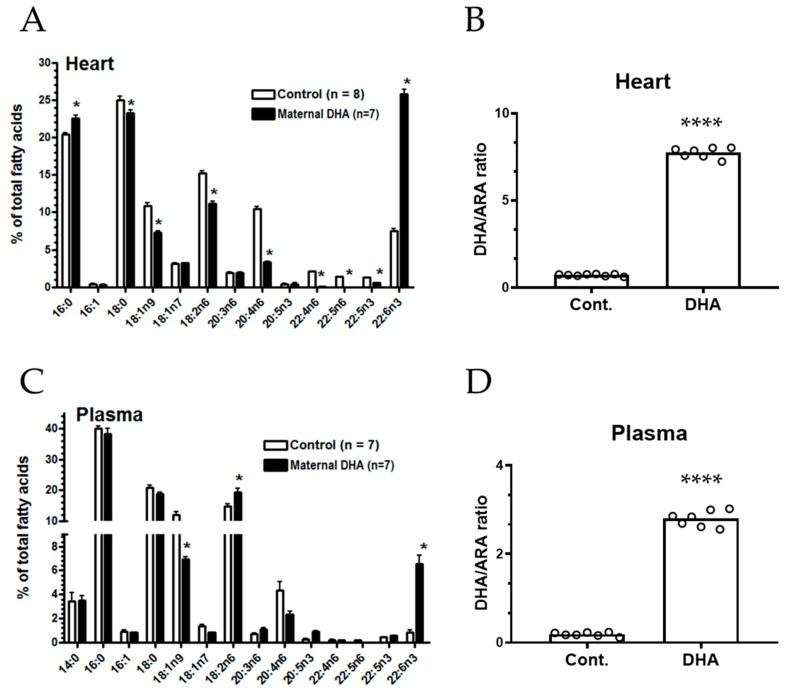
Maternal DHA intervention alters the FA composition in offspring heart and plasma. (**A**,**C**): FA composition from the heart and plasma in developing pups nursed by mothers fed a control and 1% DHA diet, respectively. (**B**,**D**): Ratios of DHA/ARA in the heart and plasma, respectively. FA composition was reported as percent of total FA content (g per 100 g). Each value represents the mean ± SEM of eight and seven pups from control and 1% DHA diet groups, respectively. Analysis of FA using one way-ANOVA indicated significance between control and 1% DHA diet group. * *p* < 0.05. Analysis of DHA/ARA ratios using two-tail unpaired t-test indicated significance between DHA group and controls. **** *p* < 0.0001.

FAs in the heart tissue from control pups are characterized by high levels of 18:2 (n-6), 20:4 (n-6), and 22:6 (n-6). Pups nursed by DHA mothers showed a large increase in 22:6 (n-3) (from 7.5 to 25.8%) and decrease in 20:4 (n-6) (from 10.5 to 3.3%) as compared to controls (Figure 5A). In addition, significant decreases in other (n-6) FA, such as 22:4 (n-6) and 22:5 (n-6) are also observed (Figure 5A). Besides the n-6 FAs, the large increase in 22:6 (n-3) is marked by a decrease in 18:2 (n-6) (15.2 to 11.2%). The DHA/ARA ratios in control and DHA pups were 7.8 versus 0.7, indicating an 11.2-fold increase (Figure 5B).

Analysis of FAs in plasma indicated high levels (14.6%) of 18:2 (n-6) but low levels (less than 1%) of 22:6 (n-3) (Figure 5C). However, plasma from the DHA pups showed a large increase in 22:6 (n-3) (from 0.8 to 6.5%) and a non-significant decrease in 20:4 (n-6) (from 4.3 to 2.3%) compared to the control pups. The large increase in 22:6 (n-3) was also marked by decreases in other FAs, e.g., a significant decrease (11.9 to 6.9%) in 18:1 (n-9). The DHA/ARA ratios in plasma were 14.9-fold higher in the DHA group compared to the control group (Figure 5D).

## 3. Discussion

**Figure 6:**
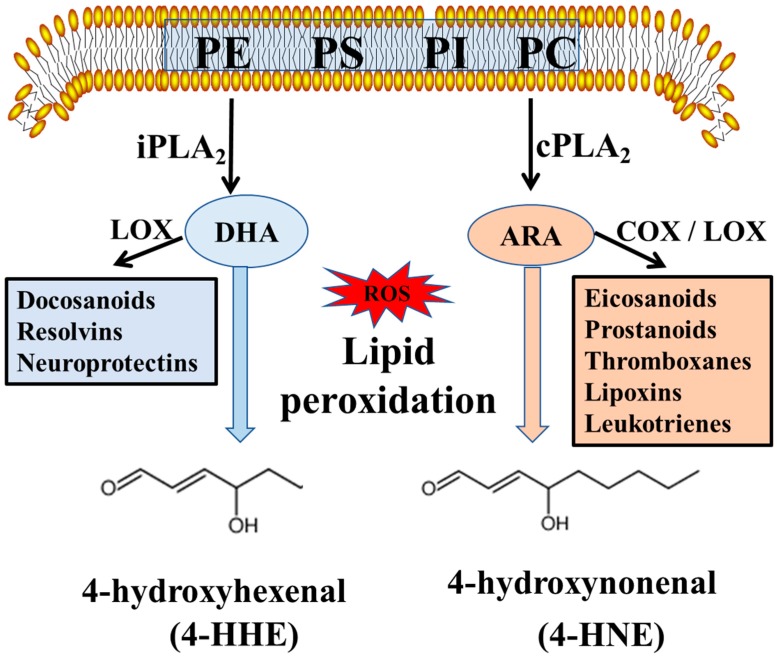
Pathways depicting release of DHA and ARA by cPLA_2_ and iPLA_2_, respectively, and subsequently metabolism to docosanoids and eicosanoids by cyclooxygenase (COX) and lipoxygenase (LOX), as well as peroxidation to form 4-HHE and 4-HNE.

In the present study, an LC-MS/MS protocol was used to determine levels of 4-HHE and 4-HNE, and gas chromatography to determine FA composition in brain regions, as well as in heart and plasma, of weanling pups born from and nursed by dams fed either a control diet or a diet supplemented with 1% DHA (Figure 1). Maternal supplementation with DHA resulted in significant increases in DHA and concomitant decreases in ARA, as well as increases in DHA/ARA ratios in all pup brain regions analyzed. However, significant increases in levels of 4-HHE, a peroxidation product of DHA, were observed mainly in the cortex and hippocampus and not in the striatum and cerebellum. Furthermore, although substantial levels of 4-HNE were present in all pup brain regions, this peroxidation product was apparently not altered by maternal dietary supplement of DHA. In our previous study, maternal DHA supplementation also resulted in an increase in DHA and decrease in ARA levels in the total pup brain [13]. Therefore, the effect of maternal DHA or n-3 PUFA to cause the increase in DHA in pup brain is not surprising, but the fact that this dietary regimen can suppress ARA, as well as n-6 PUFA is intriguing and not well understood. Recent studies demonstrated that DHA and ARA in membrane phospholipids actively undergo “deacylation-reacylation” reactions, and free ARA is released largely through the cytosolic phospholipase A_2_ (cPLA_2_), and free DHA is released through iPLA_2_ [1]. As shown in Figure 6, the cPLA_2_ pathway produces ARA which is linked to metabolites that are inflammatory, whereas the iPLA_2_ mediated production of DHA is linked to metabolites that are pro-resolving. Based on this hypothesis, increases in DHA and decreases in ARA in pups, due to maternal DHA supplementation may alter the cycle for deacylation/reacylation of membrane phospholipids and subsequently enhance the protective events and suppress the inflammatory events. Changes in these FAs may be the underlying factor for promoting neurite outgrowth and an increase in synaptic proteins, which in turn, may have an impact on learning and memory during brain development [14,31].

Endogenous levels of 4-HHE and 4-HNE are present in all brain regions as well as in the heart and plasma. Maternal DHA was shown to increase the 4-HHE levels in specific brain regions without alteration of 4-HNE levels. These results are in agreement with our earlier study with cell model indicating differences in peroxidation activities for DHA and ARA, and increase in 4-HHE upon addition of DHA, but increase in 4-HNE due mainly to stimulation of cells with lipopolysaccharide (LPS), a pathway associated with the increase in cPLA_2_ and release of ARA [32]. Consequently, studies with both cell and animal models illustrated differences in the mechanism for lipid peroxidation for DHA and ARA (Figure 6). Further studies are needed to examine whether maternal DHA alters the oxylipins in pup brains and investigate whether the enhanced production of 4-HHE may impact brain development and the neuropsychological functions of the offspring.

In contrast to the brain, analysis of FA composition in the heart muscle of pups exposed to maternal DHA indicated a large increase in DHA and a significant decrease in ARA, as well as other n-6 PUFAs. Here, the increase in DHA is linked to a large increase in 4-HHE, but similar to results in the brain, maternal DHA did not alter the 4-HNE levels in the heart. The large increase in levels of 4-HHE in the DHA pup heart clearly shows the ability for dietary DHA to modify membrane phospholipids and peroxidation products in the myocardial tissue. Since myocardial tissue is known to comprise of a large number of mitochondria, it is reasonable to surmise that the changes in the membrane FA and lipid peroxidation products in mitochondria may play a role in energy metabolism, which in turn may impact myocardial function [33]. Besides the heart, the study by Nakagawa et al. also showed changes in FA, as well as 4-HHE and 4-HNE levels in other peripheral tissues upon feeding adult mice with a fish oil-containing diet for 1 to 3 weeks [29]. However, despite changes in the peripheral tissues, study with adult mice showed only minor changes in the brain [29]. 

Our original goal to include analysis of pup plasma was to test whether changes in FAs and lipid peroxidation products in this easily accessible body fluid could serve as biomarkers for gauging effects of maternal DHA on the pup brain. Analysis of FAs in pup plasma indicated a large increase in DHA and a decrease in ARA. Interestingly, plasma from these pups not only showed large increases in 4-HHE, but also a significant decrease in the levels of 4-HNE. These results are in agreement with those reported by Nakagawa et al., who also observed significant increases in 4-HHE and decreases in 4-HNE levels in the plasma of adult mice fed with a fish oil-containing diet for 1 and 3 weeks [29]. However, the underlying mechanism for the decrease in 4-HNE in plasma but not in brain and heart remains to be elusive and not well understood. Nevertheless, despite that changes in plasma are closer to those in the heart than in the brain, analysis of FA and lipid peroxidation products in the plasma may provide useful information reflecting effects of dietary DHA on other body compartments/organs.

DHA and ARA in membrane phospholipids not only undergo metabolic turnover mediated by different phospholipases, but they are also linked to different downstream pathways providing eicosanoids which can be pro-inflammatory, and docosanoids which can be protective (Figure 6) [34,35,36]. Studies using n-3 PUFA deficiency rats indicated the important role of DHA to alter glutamatergic and serotonergic systems and enhance proliferation of neurogenesis in offspring [12]. In contrast to DHA, dietary supplementation of ARA was shown to cause an increase in inflammatory effects, including impairment of short-term memory and modifications of post-synaptic proteins [37]. With the possibility that dietary DHA can attenuate neuroinflammatory responses, more studies are needed to test whether this dietary regimen also alters microglial cell function [38,39]. 

Whether maternal DHA-mediated the increase in 4-HHE may play a role in modulating neurophysiological function is not known and needs further investigation. In studies with cell culture, lipid peroxidation products have been shown to offer adaptive benefits, and depending on the conditions, these compounds may undergo a transition from a hormetic stage to a cytotoxic stage [40,41]. It is possible that the increase in 4-HHE together with changes in the (n-3)/(n-6) FA ratios results in alteration of the redox homeostasis in the brain and heart. In a study by Zhang et al., supplementation of neurons in culture with DHA could significantly reduce neuronal death, induced by oxygen-glucose deprivation, and the protective effect was attributed to nuclear factor-like-2 (Nrf2) activation and induction of heme-oxygenase-1 (HO-1), an important anti-oxidative enzyme [22]. Mice fed with a fish oil-enhanced diet showed resistance to ischemia as compared with mice fed with a regular diet, and together with increase an in 4-HHE, the protection was also associated with upregulation of Nrf2 and production of HO-1. In the study by Nakagawa et al., adult mice fed a fish oil-containing diet also showed increases in expression of HO-1 mRNA in different organs [29]. Taken together, these studies provide strong evidence for maternal DHA to induce 4-HHE and subsequently provide adaptive changes through activation of the Nrf2 pathway. However, whether these changes play a role in reversing the neurobehavioral changes observed in the gene/stress autism animal model remains to be further examined [13].

## 4. Materials and Methods 

4-Hydroxyhexenal (4-HHE, 1 mg in 0.1 mL of ethanol), 4-hydroxynonenal (4-HNE, 1 mg in 0.1 mL of ethanol), and 4-hydroxyhexenal-d_3_ (4-HHE-d_3_, 100 μg in 0.1 mL of methyl acetate) were purchased from Cayman Chemical Co. (Ann Arbor, MI, USA). 1,3-cyclohexanedione (CHD, 97%), ammonium acetate (HPLC grade), acetic acid (ACS grade) and formic acid (mass spectrometry grade), were purchased from Sigma-Aldrich (St. Louis, MO, USA). C18 Sep-Pak cartridges (1 mL, 100 mg) were purchased from Waters Corporation (Milford, MA, USA), and phospholipid removal cartridges (Phree^TM^, 1 mL) were purchased from Phenomenex Inc. (Torrance, CA, USA). All solvents (HPLC grade) used for sample preparation, UHPLC and MS analysis were obtained from Thermo Fisher Scientific Inc. (Fair Lawn, NJ, USA).

Adult female C57BL/6J mice at age 6–8 weeks were purchased from Jackson Laboratory (Bar Harbor, ME, USA), and were fed the control diet for at least two weeks for habituation in the vivarium. Starting 2 weeks before breeding and during the entire time from breeding, through gestation and lactation, dams were fed one of two commercially-prepared mouse/rodent diets. The control diet (modified AIN-93G #103619) and an experimental diet containing 1% DHA (#103598) from Dyets Inc. (Bethlehem, PA, USA) were prepared according to the published guidelines from the American Institute of Nutrition (AIN). The composition of the control and 1% DHA diet is described in detail in Supplemental Table S3. The control diet contained no preformed DHA, but did contain sufficient amounts of alpha-linolenic acid (ALA, 18:3n-3) to meet normal brain DHA requirements [42]. Both diets were stabilized against auto-oxidation by the addition of a potent synthetic antioxidant (0.02 g tertiary-butylhydroquinone/100 g fat). The DHA source used in this study (i.e., DHASCO algal oil) contained small amounts of other long-chain n-3 PUFA ethyl esters, and was provided as a generous gift from DSM Nutritional Products, (Columbia, MD, USA). Fatty acid compositions of the control and experimental diets are shown in Supplemental Table S4.

Dietary treatments were initiated 2 weeks prior to breeding and maintained throughout gestation and lactation. At the time of weaning (21-days), 8 pups from two litters from dams fed the control diet and 7 from one litter from a dam fed the DHA diet were used. Body weight (mean ± SD) from controls and DHA pups were 8.0 ± 1.1 g (*n* = 5) and 8.5 ± 0.4 g (*n* = 7), respectively. Animals were anaesthetized with isofluorane, and blood was obtained by heart puncture. The brains were dissected to obtain left and right cerebral cortex, striatum, hippocampus and cerebellum, and stored at −80 °C. In addition, the perfused hearts were removed and stored at -80 °C. Terminal blood samples were collected by cardiac puncture following CO_2_ asphyxiation, using heparin as an anticoagulant. The blood was centrifuged at 16,100× *g* for 20 min to obtain plasma and frozen at −80 °C until use. The animal protocol was approved by the University of Missouri Animal Care and Use Committee (#8945), and performed in accordance with National Institutes of Health Animal Care and Use Guidelines.

FA analyses of plasma and tissues were carried out by direct transesterification without prior extractions as described by Lepage and Roy [43]. Briefly, small aliquots (i.e., 0.1 mL) of tissue homogenates or plasma were combined with 1 mL of methanol-benzene (3:2) containing 0.1 mg of an internal standard (C17:0 methyl ester). Then 1 mL of freshly prepared acetyl chloride-methanol (5:100, *v*/*v*) was added to the sample mixture in a glass test tube which was sealed tightly with a teflon-lined screw cap and subjected to methanolysis at 100 °C for 1 h. The samples were then cooled to room temperature, 1 mL of double-distilled water and 1 mL of hexane was added, and followed by vigorous mixing. After brief centrifugation to speed phase separation, the upper hexane layer was separated from the aqueous phase with a glass Pasteur pipette into a pre-rinsed conical glass tube through another Pasteur pipette filled with anhydrous sodium sulfate granules. Hexane was evaporated under nitrogen gas and the residue was resuspended in 100 µL of heptane. The sample was injected into the gas chromatography system. The fatty acid methyl esters were analyzed using a gas chromatograph (Agilent 7809A) equipped with a 60 m, 0.25 mm I.D., and 0.15-μm film DB-23 column. GC conditions were a helium flow rate of 1 mL/min with an initial temperature of 140 °C held for 5 min. The column temperature was then increased to 250 °C at a rate of 2 °C/min and held at 250 °C for 15 min. FAs are identified by comparison of retention times using a variety of commercial standards. FA concentrations are reported as percent of total FA content (g per 100 g).

**Figure 7:**
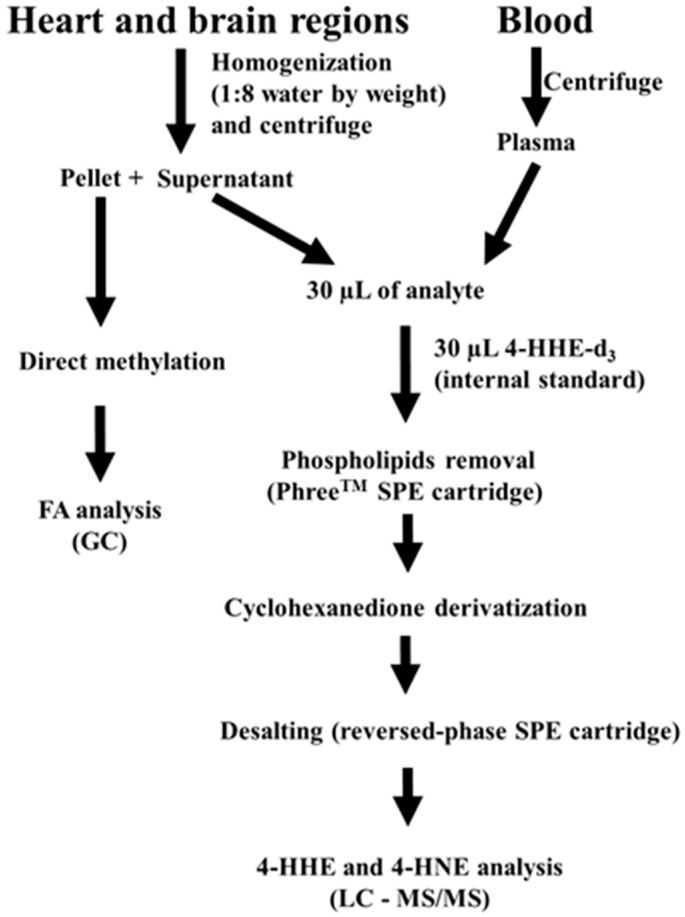
Workflow for analysis of FA, 4-HHE and 4-HNE in the offspring brain, heart and plasma.

Brain and heart samples were weighed and homogenized for 5 min in 5 or 8-fold volumes of HPLC water using a bullet blender (Next Advance, Inc., Averill Park, NY, USA) as described previously [44]. The homogenates were centrifuged at 16,100× *g* for 20 min at 4 °C, and the supernatants were collected and stored at −80 °C until analysis. A brief protocol for extraction and analysis of 4-HHE and 4-HNE from brain, heart and plasma is shown in Figure 7. LC-MS/MS analysis was carried out as described earlier [32,45]. An aliquot of supernatant was added to an equal volume of internal standard (4-HHE-d_3_, 1000 ng/mL), and acetonitrile (0.5 mL) containing 1% formic acid was added to the mixture. Solid phase extraction (SPE) was carried out using a Phree^TM^ cartridge. 4-HHE, 4-HNE and 4-HHE-d_3_ were derivatized by adding 200 µL of acidified 1,3-cyclohexanedione reagent at 60 °C for 1 h. The derivatized 4-HHE and 4-HNE were desalted using a C18 SPE cartridge and the eluate evaporated to dry. An aliquot of the reconstituted solution was injected into a Waters Xevo TQ-S triple quadrupole mass spectrometer. The multiple reaction monitoring transitions *m*/*z* 326.3 > 216.1 Da, 284.2 > 216.1 Da and 287.2 > 216.1 Da were chosen for simultaneous monitoring of 4-HNE, 4-HHE and 4-HHE-d_3_ derivatives, respectively. MassLynx software (v4.1, Waters) was used for all data acquisition. A summary of the method validation for 4-HHE and 4-HNE is shown in Supplemental Tables S5 and S6.

Statistical analyses were performed with GraphPad Prism (version 7; GraphPad Prism Software Inc., San Diego, CA, USA). Values in all figures and tables are shown as mean ± standard error of mean (SEM). Depending on the experiments, samples were analyzed by non-parametric t-tests or one-way ANOVA to compare among control and DHA diet groups. For all analyses, significance was set at *p* < 0.05. 

## 5. Conclusions

In summary, our study clearly demonstrated that maternal DHA supplementation altered FA composition together with increases of 4-HHE levels in specific brain regions. In addition, these changes were rather exaggerated in the heart and plasma of weanling pups. Expectedly, similar changes in lipid peroxidation products may occur in other tissues/body organs. In future studies, it will be important to investigate whether such changes are associated with specific cell types or sub-regions, and whether the changes in peroxidation products reflect alterations of redox activity, as well as subsequent behavioral and cognitive functions in the brain. In addition, since changes were resulted from introducing dietary DHA to pregnant mothers and throughout gestation and lactation period, it may be of interest to perform future studies to test effects of DHA supplement to dams during gestation or lactation period.

## Supplementary Materials

The following are available online at https://www.mdpi.com/2218-1989/9/3/40/s1, Table S1. Fatty acid (FA) composition of the brain (cortex, striatum, hippocampus and cerebellum) from weaning mice reared by dams fed control or DHA-supplemented (1%, *w*/*w*) diets. Table S2. Fatty Acid (FA) composition of plasma and heart from weaning mice reared by dams fed control or DHA-supplemented (1%, *w*/*w*) diets. Table S3. Experimental mouse diets. Table S4. Fatty acid composition of diets. Table S5. Method validation parameters for detection of 4-HHE in plasma, heart and brain tissue. Table S6. Method validation parameters for detection of 4-HNE in plasma, heart and brain tissue.

## References

[1] Sun G.Y., Simonyi A., Fritsche K.L., Chuang D.Y., Hannink M., Gu Z., Greenlief C.M., Yao J.K., Lee J.C., Beversdorf D.Q. (2018). Docosahexaenoic acid (DHA): An essential nutrient and a nutraceutical for brain health and diseases. Prostaglandins Leukot. Essent. Fat. Acids.

[2] Yavin E., Glozman S., Green P. (2001). Docosahexaenoic acid sources for the developing brain during intrauterine life. Nutr. Health.

[3] Bradbury J. (2011). Docosahexaenoic acid (DHA): An ancient nutrient for the modern human brain. Nutrients.

[4] Tang M., Zhang M., Wang L., Li H., Cai H., Dang R., Jiang P., Liu Y., Xue Y., Wu Y. (2018). Maternal dietary of n-3 polyunsaturated fatty acids affects the neurogenesis and neurochemical in female rat at weaning. Prostaglandins Leukot. Essent. Fat. Acids.

[5] Lyall K., Munger K.L., O’Reilly E.J., Santangelo S.L., Ascherio A. (2013). Maternal dietary fat intake in association with autism spectrum disorders. Am. J. Epidemiol..

[6] Ooi Y.P., Weng S.J., Kossowsky J., Gerger H., Sung M. (2017). Oxytocin and Autism Spectrum Disorders: A Systematic Review and Meta-Analysis of Randomized Controlled Trials. Pharmacopsychiatry.

[7] Keim S.A., Gracious B., Boone K.M., Klebanoff M.A., Rogers L.K., Rausch J., Coury D.L., Sheppard K.W., Husk J., Rhoda D.A. (2018). omega-3 and omega-6 Fatty Acid Supplementation May Reduce Autism Symptoms Based on Parent Report in Preterm Toddlers. J. Nutr..

[8] Julvez J., Mendez M., Fernandez-Barres S., Romaguera D., Vioque J., Llop S., Ibarluzea J., Guxens M., Avella-Garcia C., Tardon A. (2016). Maternal Consumption of Seafood in Pregnancy and Child Neuropsychological Development: A Longitudinal Study Based on a Population With High Consumption Levels. Am. J. Epidemiol..

[9] James S., Montgomery P., Williams K. (2011). Omega-3 fatty acids supplementation for autism spectrum disorders (ASD). Cochrane Database Syst. Rev..

[10] Horvath A., Lukasik J., Szajewska H. (2017). omega-3 Fatty Acid Supplementation Does Not Affect Autism Spectrum Disorder in Children: A Systematic Review and Meta-Analysis. J. Nutr..

[11] Mankad D., Dupuis A., Smile S., Roberts W., Brian J., Lui T., Genore L., Zaghloul D., Iaboni A., Marcon P.M. (2015). A randomized, placebo controlled trial of omega-3 fatty acids in the treatment of young children with autism. Mol. Autism.

[12] Tang M., Zhang M., Cai H., Li H., Jiang P., Dang R., Liu Y., He X., Xue Y., Cao L. (2016). Maternal diet of polyunsaturated fatty acid altered the cell proliferation in the dentate gyrus of hippocampus and influenced glutamatergic and serotoninergic systems of neonatal female rats. Lipids Health Dis..

[13] Matsui F., Hecht P., Yoshimoto K., Watanabe Y., Morimoto M., Fritsche K., Will M., Beversdorf D. (2017). DHA Mitigates Autistic Behaviors Accompanied by Dopaminergic Change in a Gene/Prenatal Stress Mouse Model. Neuroscience.

[14] Cao D., Kevala K., Kim J., Moon H.S., Jun S.B., Lovinger D., Kim H.Y. (2009). Docosahexaenoic acid promotes hippocampal neuronal development and synaptic function. J. Neurochem..

[15] Asatryan A., Bazan N.G. (2017). Molecular mechanisms of signaling via the docosanoid neuroprotectin D1 for cellular homeostasis and neuroprotection. J. Biol. Chem..

[16] Chiurchiu V., Leuti A., Dalli J., Jacobsson A., Battistini L., Maccarrone M., Serhan C.N. (2016). Proresolving lipid mediators resolvin D1, resolvin D2, and maresin 1 are critical in modulating T cell responses. Sci. Transl. Med..

[17] Jones K.L., Smith R.M., Edwards K.S., Givens B., Tilley M.R., Beversdorf D.Q. (2010). Combined effect of maternal serotonin transporter genotype and prenatal stress in modulating offspring social interaction in mice. Int. J. Dev. Neurosci..

[18] Jones K.L., Will M.J., Hecht P.M., Parker C.L., Beversdorf D.Q. (2013). Maternal diet rich in omega-6 polyunsaturated fatty acids during gestation and lactation produces autistic-like sociability deficits in adult offspring. Behav. Brain Res..

[19] Weiser M.J., Mucha B., Denheyer H., Atkinson D., Schanz N., Vassiliou E., Benno R.H. (2016). Dietary docosahexaenoic acid alleviates autistic-like behaviors resulting from maternal immune activation in mice. Prostaglandins Leukot. Essent. Fat. Acids.

[20] Guichardant M., Bacot S., Moliere P., Lagarde M. (2006). Hydroxy-alkenals from the peroxidation of n-3 and n-6 fatty acids and urinary metabolites. Prostaglandins Leukot. Essent. Fat. Acids.

[21] Lee W.C., Wong H.Y., Chai Y.Y., Shi C.W., Amino N., Kikuchi S., Huang S.H. (2012). Lipid peroxidation dysregulation in ischemic stroke: Plasma 4-HNE as a potential biomarker?. Biochem. Biophys. Res. Commun..

[22] Zhang M., Wang S., Mao L., Leak R.K., Shi Y., Zhang W., Hu X., Sun B., Cao G., Gao Y. (2014). Omega-3 fatty acids protect the brain against ischemic injury by activating Nrf2 and upregulating heme oxygenase 1. J. Neurosci..

[23] Moniuszko-Malinowska A., Luczaj W., Jarocka-Karpowicz I., Pancewicz S., Zajkowska J., Andrisic L., Zarkovic N., Skrzydlewska E. (2016). Lipid peroxidation in the pathogenesis of neuroborreliosis. Free Radic. Biol. Med..

[24] Luczaj W., Moniuszko A., Jarocka-Karpowicz I., Pancewicz S., Andrisic L., Zarkovic N., Skrzydlewska E. (2016). Tick-borne encephalitis--lipid peroxidation and its consequences. Scand. J. Clin. Lab. Investig..

[25] Luczaj W., Gegotek A., Skrzydlewska E. (2017). Antioxidants and HNE in redox homeostasis. Free Radic. Biol. Med..

[26] Csala M., Kardon T., Legeza B., Lizak B., Mandl J., Margittai E., Puskas F., Szaraz P., Szelenyi P., Banhegyi G. (2015). On the role of 4-hydroxynonenal in health and disease. Biochim. Biophys. Acta.

[27] Hu C., Wang M., Han X. (2017). Shotgun lipidomics in substantiating lipid peroxidation in redox biology: Methods and applications. Redox Biol..

[28] Long E.K., Picklo M.J. (2010). Trans-4-hydroxy-2-hexenal, a product of n-3 fatty acid peroxidation: Make some room HNE. Free Radic. Biol. Med..

[29] Nakagawa F., Morino K., Ugi S., Ishikado A., Kondo K., Sato D., Konno S., Nemoto K., Kusunoki C., Sekine O. (2014). 4-Hydroxyhexenal derived from dietary n-3 polyunsaturated fatty acids induces anti-oxidative enzyme heme oxygenase-1 in multiple organs. Biochem. Biophys. Res. Commun..

[30] Sun G.Y., Horrocks L.A. (1968). The fatty acid and aldehyde composition of the major phospholipids of mouse brain. Lipids.

[31] Calderon F., Kim H.Y. (2004). Docosahexaenoic acid promotes neurite growth in hippocampal neurons. J. Neurochem..

[32] Yang B., Li R., Greenlief C.M., Fritsche K.L., Gu Z., Cui J., Lee J.C., Beversdorf D.Q., Sun G.Y. (2018). Unveiling anti-oxidative and anti-inflammatory effects of docosahexaenoic acid and its lipid peroxidation product on lipopolysaccharide-stimulated BV-2 microglial cells. J. Neuroinflamm..

[33] Roy J., Fauconnier J., Oger C., Farah C., Angebault-Prouteau C., Thireau J., Bideaux P., Scheuermann V., Bultel-Ponce V., Demion M. (2017). Non-enzymatic oxidized metabolite of DHA, 4(RS)-4-F4t-neuroprostane protects the heart against reperfusion injury. Free Radic. Biol. Med..

[34] Bazan N.G. (2007). Omega-3 fatty acids, pro-inflammatory signaling and neuroprotection. Curr. Opin. Clin. Nutr. Metab. Care.

[35] Mukherjee P.K., Chawla A., Loayza M.S., Bazan N.G. (2007). Docosanoids are multifunctional regulators of neural cell integrity and fate: Significance in aging and disease. Prostaglandins Leukot. Essent. Fat. Acids.

[36] Serhan C.N. (2006). Novel chemical mediators in the resolution of inflammation: Resolvins and protectins. Anesthesiol. Clin..

[37] Thomas M.H., Paris C., Magnien M., Colin J., Pelleieux S., Coste F., Escanye M.C., Pillot T., Olivier J.L. (2017). Dietary arachidonic acid increases deleterious effects of amyloid-beta oligomers on learning abilities and expression of AMPA receptors: Putative role of the ACSL4-cPLA2 balance. Alzheimer’s Res. Ther..

[38] Hopperton K.E., Trepanier M.O., James N.C.E., Chouinard-Watkins R., Bazinet R.P. (2018). Fish oil feeding attenuates neuroinflammatory gene expression without concomitant changes in brain eicosanoids and docosanoids in a mouse model of Alzheimer’s disease. Brain Behav. Immun..

[39] Hopperton K.E., Trepanier M.O., Giuliano V., Bazinet R.P. (2016). Brain omega-3 polyunsaturated fatty acids modulate microglia cell number and morphology in response to intracerebroventricular amyloid-beta 1-40 in mice. J. Neuroinflamm..

[40] Chen Z.H., Yoshida Y., Saito Y., Noguchi N., Niki E. (2006). Adaptive response induced by lipid peroxidation products in cell cultures. FEBS Lett..

[41] Cohen G., Riahi Y., Sunda V., Deplano S., Chatgilialoglu C., Ferreri C., Kaiser N., Sasson S. (2013). Signaling properties of 4-hydroxyalkenals formed by lipid peroxidation in diabetes. Free Radic. Biol. Med..

[42] Domenichiello A.F., Kitson A.P., Bazinet R.P. (2015). Is docosahexaenoic acid synthesis from alpha-linolenic acid sufficient to supply the adult brain?. Prog. Lipid Res..

[43] Lepage G., Roy C.C. (1984). Improved recovery of fatty acid through direct transesterification without prior extraction or purification. J. Lipid Res..

[44] Johnson M.C., Song H., Cui J., Mossine V.V., Gu Z., Greenlief C.M. (2016). Development of a Method and Validation for the Quantitation of FruArg in Mice Plasma and Brain Tissue Using UPLC-MS/MS. ACS Omega.

[45] Sun G.Y., Li R., Yang B., Fritsche K.L., Beversdorf D.Q., Lubahn D.B., Geng X., Lee J.C., Greenlief C.M. (2019). Quercetin Potentiates Docosahexaenoic Acid to Suppress Lipopolysaccharide-induced Oxidative/Inflammatory Responses, Alter Lipid Peroxidation Products, and Enhance the Adaptive Stress Pathways in BV-2 Microglial Cells. Int. J. Mol. Sci..

