# Supplementary material for: Maternal Dietary Docosahexaenoic Acid Alters Lipid Peroxidation Products and (n-3)/(n-6) Fatty Acid Balance in Offspring Mice
Source: Metabolites. 2019 Mar 1;9(3):40. doi: 10.3390/metabo9030040 (PMC6468482; doi:10.3390/metabo9030040)
Supplement: Supplementary file 1 [file metabolites-09-00040-s001.pdf]

SUPPLEMENTAL INFORMATION:

Article

Maternal dietary docosahexaenoic acid alters lipid peroxidation products and (n-3)/(n-6) fatty acid balance in offspring mice

Bo Yang [1], Runting Li [2], Taeseon Woo [3], Jimmy D. Browning Jr. [4], Hailong Song [5], Zezong Gu [5], Jiankun Cui [5], James C. Lee [6], Kevin L. Fritsche [4], David Q. Beversdorf [3], Grace Y. Sun [2], and C. Michael Greenlief [1,*]

[1] Department of Chemistry, University of Missouri, Columbia, MO 65211, USA; BY: bykm5@mail.missouri.edu, CMG: greenliefm@missouri.edu
[2] Biochemistry Department, University of Missouri, Columbia, MO 65211, USA; RL: lirun@health.missouri.edu, GYS: sung@missouri.edu
[3] Departments of Radiology, Neurology and Psychological Sciences, and the Thompson Center, University of Missouri, Columbia, MO 65211, USA; TW: twfnf@mail.missouri.edu, DQB: beversdorfd@health.missouri.edu
[4] Department of Nutrition and Exercise Physiology, University of Missouri, Columbia, MO 65211, USA; JDB: browningj@missouri.edu, KLF: fritschek@missouri.edu
[5] Department of Pathology and Anatomical Sciences, University of Missouri, Columbia, MO 65211, USA; HS: hskk8@mail.missouri.edu, ZG: guze@health.missouri.edu, JC: cuij@health.missouri.edu
[6] Department of Bioengineering, University of Illinois at Chicago, Chicago, IL 60607, USA; leejam@uic.edu
* Correspondence: greenliefm@missouri.edu; Tel.: +01-573-882-3288

Table S1. Fatty Acid (FA) Composition of Brain (Cortex, Striatum, Hippocampus and Cerebellum) from Weaning Mice Reared by Dams Fed Control or DHA-Supplemented (1%, w/w) Diets

FA	Cortex		Striatum		Hippocampus		Cerebellum	
	Control	DHA	Control	DHA	Control	DHA	Control	DHA
14:0	nd[a]	nd	nd	nd	nd	nd	nd	nd
16:0	29.4 ± 0.3	29.6 ± 0.2	26.4 ± 0.6	26.1 ± 0.9	25.6 ± 0.7	25.6 ± 0.7	21.8 ± 0.1	22.2 ± 0.3
16:1	0.7 ± 0.02	0.7 ± 0.01	0.7 ± 0.01	0.7 ± 0.02	0.6 ± 0.02	0.6 ± 0.02	0.7 ± 0.02	0.7 ± 0.01
18:0	28.2 ± 0.2	28.6 ± 0.1	27.1 ± 0.5	27.4 ± 0.5	25.6 ± 0.5	25.5 ± 0.4	22.1 ± 0.2	22.4 ± 0.4
18:1(n-9)	12.7 ± 0.1	13.0 ± 0.1	16.4 ± 0.4	16.6 ± 0.5	12.7 ± 0.2	13.2 ± 0.2	18.2 ± 0.2	18.7 ± 0.2
18:1(n-7)	3.2 ± 0.02	3.0 ± 0.02	4.0 ± 0.1	3.7 ± 0.2	3.0 ± 0.04	2.8 ± 0.03	4.5 ± 0.03	4.3 ± 0.06
18:2(n-6)	0.9 ± 0.02	1.1 ± 0.01	0.9 ± 0.03	1.1 ± 0.05	0.8 ± 0.02	1.0 ± 0.02	1.4 ± 0.03	1.7 ± 0.03
20:3(n-6)	***0.5 ± 0.02***	***1.0 ± 0.01***	0.7 ± 0.04	1.2 ± 0.05	0.6 ± 0.03	1.1 ± 0.04	1.0 ± 0.01	1.5 ±0.03
20:4(n-6)	***10.5 ± 0.1***	***8.8 ± 0.1***	***9.6 ± 0.1***	***8.2 ± 0.4***	***13.1 ± 0.4***	***11.1 ± 0.3***	***9.9 ± 0.2***	***7.4 ± 0.1***
20:5(n-3)	nd	nd	nd	nd	nd	0.2 ± 0.02	nd	0.2 ± 0.01
22:4(n-6)	***2.2 ± 0.04***	***1.2 ± 0.02***	***2.6 ± 0.1***	***1.6 ± 0.1***	2.6 ± 0.06	1.5 ± 0.06	***2.7 ± 0.08***	***1.4 ± 0.06***
22:5(n-6)	***0.6 ± 0.02***	***nd***	0.6 ± 0.02	nd	0.8 ± 0.03	0.1 ± 0.03	0.4 ± 0.02	nd
22:5(n-3)	0.3 ± 0.03	0.4 ± 0.03	0.9 ± 0.06	0.9 ± 0.1	0.4 ± 0.05	0.4 ± 0.02	1.8 ± 0.06	2.1 ± 0.1
22:6(n-3)	***10.7 ± 0.4***	***12.7 ± 0.2***	***10.1 ± 0.4***	***12.3 ± 0.8***	***14.2 ± 0.5***	***16.9 ± 0.8***	***15.6 ± 0.2***	***17.6 ± 0.3***
n-3: n-6 ratio	0.75	1.08	0.76	1.09	0.82	1.18	1.13	1.66

[a] "nd", not detected.

Values shown in italics and bold for FA compositions are significantly different between the control and DHA group.

Table S2. Fatty Acid (FA) Composition of Plasma and Heart from Weaning Mice Reared by Dams Fed Control or DHA-Supplemented (1%, w/w) Diets

FA	Plasma		Heart	
	Control	DHA	Control	DHA
14:0	3.5 ± 0.7	3.5 ± 0.4	nd	nd
16:0	40.0 ± 0.7	38.3 ± 1.6	***20.4 ± 0.3***	***22.6 ± 0.3***
16:1	0.9 ± 0.1	0.8 ± 0.1	0.4 ± 0.05	0.3 ± 0.05
18:0	20.9 ± 0.6	18.7 ± 0.6	***25.0 ± 0.6***	***23.2 ± 0.4***
18:1 (n-9)	***11.9 ± 1.1***	***6.9 ± 0.2***	***10.9 ± 0.4***	***7.3 ± 0.2***
18:1 (n-7)	1.4 ± 0.1	0.8 ± 0.04	3.2 ± 0.1	3.2 ± 0.05
18:2 (n-6)	***14.6 ± 0.8***	***19.3 ± 1.3***	***15.2 ± 0.3***	***11.2 ± 0.3***
20:3 (n-6)	0.7 ± 0.07	1.1 ± 0.1	1.9 ± 0.1	1.9 ± 0.04
20:4 (n-6)	4.3 ± 0.7	2.3 ± 0.3	***10.5 ± 0.3***	***3.3 ± 0.1***
20:5 (n-3)	0.2 ± 0.05	0.2 ± 0.1	0.3 ± 0.1	0.4 ± 0.2
22:4 (n-6)	0.3 ± 0.01	0.9 ± 0.05	***2.1 ± 0.1***	***0.1 ± 0.05***
22:5 (n-6)	0.2 ± 0.01	nd[a]	***1.4 ± 0.04***	***nd***
22:5 (n-3)	0.4 ± 0.03	0.5 ± 0.02	1.3 ± 0.1	0.6 ± 0.05
22:6 (n-3)	***0.8 ± 0.2***	***6.5 ± 0.7***	***7.5 ± 0.3***	***25.8 ± 0.6***
n-3: n-6 ratio	0.07	0.31	***0.29***	***1.62***

[a] "nd", not detected.

Values shown in italics and bold for FA compositions are significantly different between the control and DHA group.

Table S3. Experimental Mouse Diets

Ingredients (g / kg)	Control diet	1% DHA diet
cornstarch	397	397
casein	200	200
dextrin (dyetrose)	132	132
sucrose	100	100
fiber (alpha-cellulose)	50	50
mineral mix (AIN-93)	35	35
vitamin mix (AIN-93G)	10	10
L-cystine	3	3
choline bitartrate	2.5	2.5
soybean oil	50	0
corn oil	20	0
safflower oil	0	45
DHASCO algal oil[a]	0	25
t-butylhydroquinone (a synthetic anti-oxidant)	0.014	0.014
total	1000	1000

[a]a gift from DSM Nutritional Products, Columbia, MD, USA

Table S4. Fatty Acid Composition of Diets

	Control diet	1% DHA diet
16:0	0.8[a]	0.4
18:0	0.2	0.2
18:1	1.8	1.0
18:2n6	3.9	4.0
18:3n3	0.4	0.1
20:5n3	nd[b]	0.1
22:5n3	nd	0.3
22:6n3 (DHA)	nd	1.0

[a] Values represent g/ 100g of diet.

[b] nd: not detected.

Table S5. Method Validation Parameters for Detection of 4-HHE in Plasma, Heart and Brain Tissue

	Linearity	Matrix Effect	Phree Recovery	Sep-Pak Recovery	Intra-day		Inter-day	
					Accuracy (bias)	Precision (CV[a])	Accuracy (bias)	Precision (CV[a])
Plasma (n = 3)	0.9986	100.7%	109.9%	100.1%	9.7%	0.8%	11.9%	0.9%
Heart (n =3)	0.9995	89.6%	91.2%	99.8%	6.5%	0.6%	7.2%	0.7%
Brain (n = 3)	0.9969	98.1%	95.3%	110.1%	7.4%	1.8%	13.6%	2.1%

[a] CV: coefficient of variance. “n” represents the number of a given biological sample and triplicate analyses were obtained for each biological sample.

Table S6. Method Validation Parameters for Detection of 4-HNE in Plasma, Heart and Brain Tissue

	Linearity	Matrix Effect	Phree Recovery	Sep-Pak Recovery	Intra-day		Inter-day	
					Accuracy (bias)	Precision (CV[a])	Accuracy (bias)	Precision (CV[a])
Plasma (n = 3)	0.9993	100.7%	88.4%	95.2%	8.6%	1.7%	14.3%	2.2%
Heart (n = 3)	0.9967	89.6%	89.9%	94.9%	5.6%	1.4%	10.8%	3.0%
Brain (n = 3)	0.9932	98.1%	85.6%	111.7%	6.6%	2.0%	12.7%	6.4%

[a] CV: coefficient of variance. "n" represents the number of a given biological sample and triplicate analyses were obtained for each biological sample.
